# Cannabinoid signaling and risk in Huntington's disease

**DOI:** 10.3389/fncom.2022.903947

**Published:** 2022-09-02

**Authors:** James Humble, James R. Kozloski

**Affiliations:** Health Care and Life Sciences, T. J. Watson IBM Research Center, Yorktown Heights, NY, United States

**Keywords:** endocannabinoid, Huntington's disease (HD), metabolic risk, computational model, excitotoxicity

## Abstract

Dysregulated endocannabinoid (eCB) signaling and the loss of cannabinoid receptors (*CB*1*R*s) are important phenotypes of Huntington's disease (HD) but the precise contribution that eCB signaling has at the circuit level is unknown. Using a computational model of spiking neurons, synapses, and eCB signaling, we demonstrate that eCB signaling functions as a homeostatic control mechanism, minimizing excess glutamate. Furthermore, our model demonstrates that metabolic risk, quantified by excess glutamate, increases with cortico-striatal long-term depression (LTD) and/or increased cortico-striatal activity, and replicates a progressive loss of cannabinoid receptors on inhibitory terminals as a function of the excitatory/inhibitory ratio.

## Introduction

Endocannabinoid (eCB) signaling is prevalent throughout the central and peripheral nervous systems, and in the central nervous system, it is found on both excitatory and inhibitory terminals (Cachope, 2012). Several mechanisms of eCB signaling and regulation have been observed, and we review them here to motivate our quantitative and computational model and a broader review of the systems neurobiology of eCB (Lu and Mackie, 2021). One commonly observed form of retrograde eCB signaling induces long-term depression (LTD) at synapses, termed eCB-LTD, and is thought to function homeostatically (Cachope, 2012). Endocannabinoids released from the post-synaptic site activate eCB receptors (CB1R) on the pre-synaptic site. CB1R activation then suppresses the release of neurotransmitters *via* Ca^2+^ channel inhibition (Maejima et al., 2001). eCB-LTD has been found throughout the brain, including in the hippocampus (Shen et al., 1996) and the striatum (Gerdeman and Lovinger, 2001). In the striatum, eCB signaling is present on both the cortico-striatal synapse and the intra-striatal inhibitory synapses (Narushima et al., 2006) and minimally on thalamo-striatal terminals (Wu et al., 2015).

Several different mechanisms have been observed for the production and release of eCBs (Younts and Castillo, 2013; Heifets and Castillo, 2015). One form of eCB signaling releases eCBs from spines and dendrites, where they are produced on demand in an activity-dependent manner. In a spine, both the activation of a metabotropic glutamate receptor 5 (mGluR5s) and an intra-spine increase in Ca^2+^ are typically required for eCB production. However, in a dendrite, an increase in intracellular Ca^2+^ alone is sufficient (Kano et al., 2009).

Huntington's disease (HD) is a neurodegenerative disease with a progressive decline in motor and cognitive function. It is caused by the expansion of a cytosine-adenine-guanine repeat within exon 1 of the huntingtin gene (MacDonald et al., 1993). One of the earliest observed changes in both patients with HD and HD animal models is eCB system dysregulation. Striatal neurons degenerate (Glass et al., 2000) and CB1Rs are progressively lost throughout the striatum. Receptor deletion *via* a double-mutant mouse model further aggravates HD symptoms (Blázquez et al., 2011). R6/1 mice (with 115 CAG repeats) show a 27% decrease in CB1 messenger ribonucleic acid (mRNA) in the striatum and a 19% decrease in protein in the substantia nigra, with CB1R ligand binding reduced by 20% in many basal ganglia regions (Dowie et al., 2009).

A primary hypothesis involves an initial overactivity of glutamate terminals, possibly due to a dysfunction in glutamate transporter-1 (GLT1), which is responsible for glutamate uptake (Liévens et al., 2001; Estrada-Sánchez and Rebec, 2012), leading to excitotoxicity. Subsequently, other changes are seen in the striatum and neocortex (Bari et al., 2013), with simultaneous changes in dopamine observed (André et al., 2010). Furthermore, excitotoxic damage induced by administering quinolinic acid to normal animals and those that selectively lack CB1R on cortico-striatal glutamatergic synapses or on striatal gamma-aminobutyric acid (GABA)ergic neurons demonstrated that normal eCB signaling on the glutamatergic synapses plays a key neuroprotective role (Chiarlone et al., 2014).

The dysfunction of eCB signaling observed in HD models is not constant across all terminals. Instead, among R6/2 HD mice (which express *exon 1* of the human HD gene), the sensitivity of GABA synapses to eCBs is impaired in the striatum (Chiodi et al., 2011), while glutamate signaling retains sensitivity to eCB (Centonze et al., 2005). Furthermore, CB1 signaling is downregulated in medium spiny neurons (MSNs) of the indirect pathway, and CB1R expression is decreased in neuropeptide Y/neuronal nitric oxide synthase (NPY/nNOS)-expressing interneurons (Horne et al., 2013), with Caudate-Putamen to globus pallidus (GP) terminals still demonstrating eCB suppression (Engler et al., 2005). A loss of parvalbuminergic inhibitory interneurons has been observed in the postmortem tissue of patients with HD (Reiner et al., 2013), and enhanced feedforward synaptic activity is observed in HD mouse models (Cepeda et al., 2013).

In addition to these various dysfunctions of eCB signaling, a common theme in HD is synaptic disconnection (Barry et al., 2021). Disconnection is more prevalent among excitatory terminals, with a loss of both cortico- and thalamo-striatal excitatory terminals in the Q140 (with 140 CAG repeats) knock-in HD mouse model (Deng et al., 2014). CB1 is differentially expressed in normal animals at cortico-striatal, thalamo-striatal, and intra-striatal terminals within associative and limbic pathways vs. sensorimotor pathways (Waes et al., 2012; Wu et al., 2015). The differential loss of CB1R in GP externus (GPe) and GP internus (GPi) results in an upregulation of GABA receptors in the GP, and it has been suggested that this upregulation may be compensatory (Allen et al., 2009).

Data are not limited to animal models—human positron emission tomography (PET) studies have also observed a decrease in CB1R availability in premanifest HD and a further progressive decrease in disease burden (Laere et al., 2010; Ceccarini et al., 2016).

The precise contribution that eCB signaling has at the circuit level, if any, is unknown and there have been no computational studies of eCB signaling at a network/circuit model level. To explore eCB signaling at this level, we began by modeling a population of spiking neurons and synapses with eCB signaling present. In the model, we found that eCB signaling can function as a homeostatic controller of glutamatergic terminals. As such, the release of glutamate is adjusted by running the model such that glutamate released equals the amount required to activate α-amino-3-hydroxy-5-methyl-4-isoxazolepropionic acid (AMPA) receptors, plus an additional amount needed to activate mGluR5.^1^ At GABAergic terminals, the model produced a similar, albeit less strongly regulated, phenomenon. We further used the model to quantify metabolic risk across different neurons in the population under different perturbation conditions and circuit setpoints. Finally, we explored changes to the excitatory/inhibitory balance in the model and found that CB1R can be lost with a decrease in this ratio when we introduced a proposed process *X* into the model.

## Results

To explore the ability of eCB signaling to function in our model as a homeostatic mechanism, we initialized our simulations with random values for neurotransmitter release, AMPAR number, GABAR number, and CB1R number, and ran them until the system stabilized. We report results from this stabilization period throughout. For each simulation, we observed that varying kinetics and parameters of the various eCB signaling components resulted in a period of mismatch between the amount of glutamate or GABA released and the amount needed to bind the number of AMPAR or GABAR in the model. We quantified this mismatch at each time point and termed it “excess glutamate/GABA.” “Excess” in our results can be positive or negative, with positive representing the release of more neurotransmitters than required to bind all receptors and negative representing less. For example, if a glutamatergic bouton releases 0.6 a.u. of glutamate, and the spine's number of AMPAR is 0.2 a.u., then there is excess glutamate of 0.4 a.u. However, if a GABAergic bouton releases 0.4 a.u. of GABA, and the bouton's number of GABAR is 0.7 a.u., there is excess GABA of −0.3 a.u.

**Figure 1:**
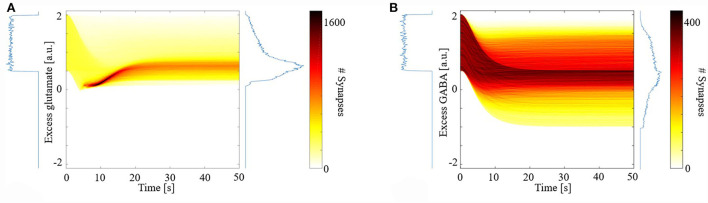
Distribution of synaptic excess glutamate **(A)** and gamma-aminobutyric acid (GABA) **(B)** as a function of simulation time.

Figure 1 shows the distribution of excess neurotransmitters over all synapses as a function of simulation time. For both glutamate (Figure 1A) and GABA (Figure 1B), initial excess neurotransmitter distributions are uniform due to the chosen distribution for random parameter initialization. Over the ensuing time, however, these distributions undergo transformation by the model's different eCB signaling components and their kinetics. All distributions were finally stabilized by 50 s, when all simulations ended. In the final steady state, eCB signaling had homeostatically controlled the release of glutamate such that few terminals released large excess glutamate, and most released small excess glutamate. We further observed that the model maintained these small excesses and did not suppress them to zero. In the model, this excess amount is required to bind to mGluR5s and N-methyl-D-aspartate receptors (NMDARs) in the spines, in turn, the activation of which fulfills the two pre-requisites for modeled eCB production. This strong homeostatic maintenance was not observed in the model of GABAergic terminals. Instead, the distribution of excess GABA across all synapses after model stabilization was broad and long-tailed, indicating that many synapses continued to release large excess GABA and many large negative excesses, with fewer releasing close to the amount required to bind all GABARs. The required excess amount of glutamate is an interesting prediction of the model and requires experimental verification.

**Figure 2:**
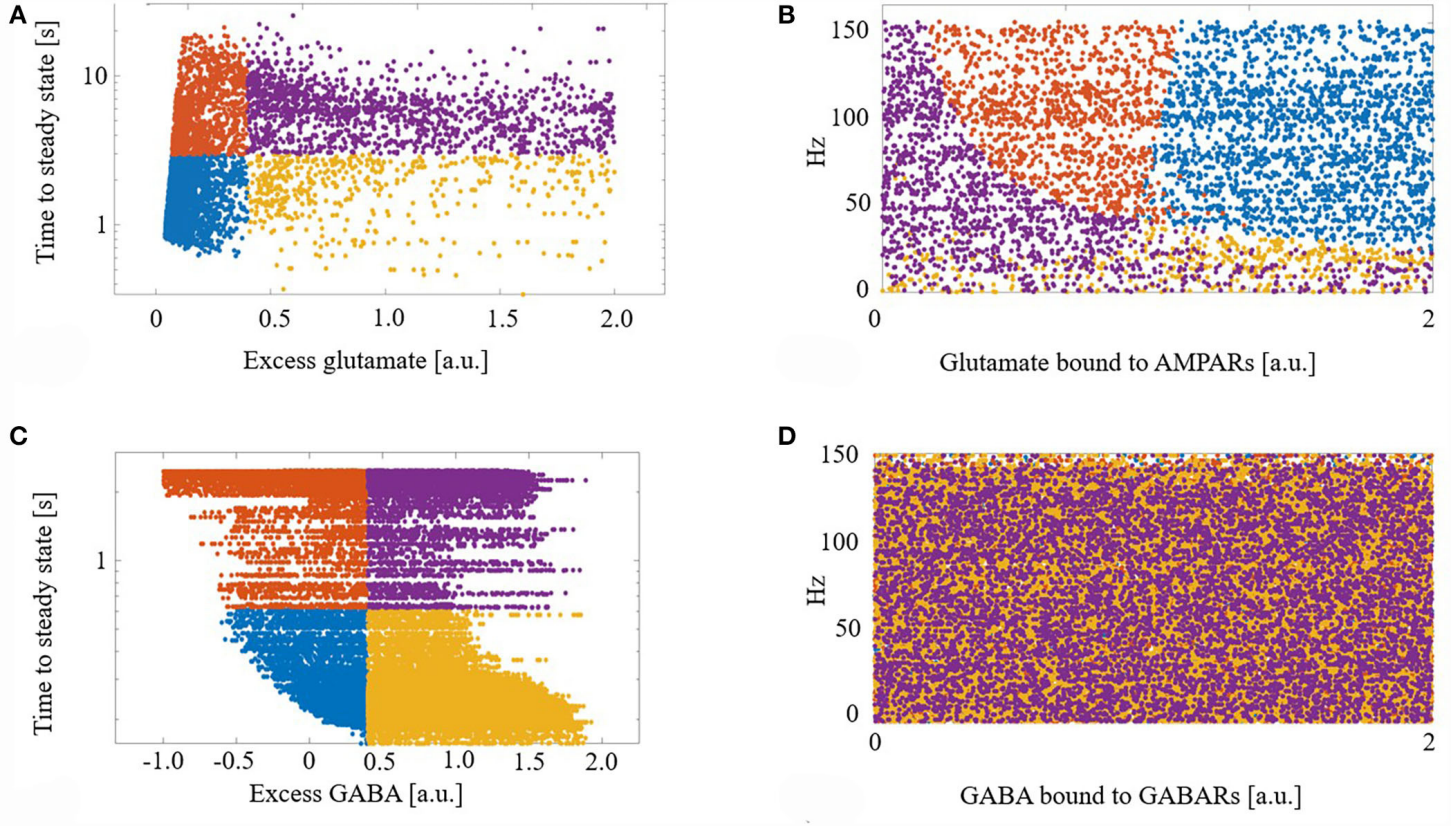
**(A)** Demarcation of excess glutamate dynamics into the four synapse groups at the median excess glutamate and the median time to steady state: (blue) timely maintained with a strong control of excess glutamate, (yellow) timely maintained but with a weak control of excess glutamate, (orange) untimely maintained with a strong control of excess glutamate, and (purple) untimely maintained with a weak control of excess glutamate. **(B)** The four groups are still segregated in a new projected space of glutamate bound to AMPAR and driving excitatory firing rate. **(C,D)** As in **(A,B)** for GABA. **(D)** The four groups are lost when projected into the space of GABA bound to GABA receptors (GABARs) and the excitatory firing rate.

We next explored the dynamics of this excess neurotransmitter. For each synapse, excess neurotransmitters changed monotonically until achieving a steady state. We measured the time to steady state for each synapse, defined as the time at which a synapse's excess neurotransmitter was within one standard deviation (SD) of the synapse's mean excess over the final 5 s of a simulation. For each synapse, we also measured its final steady state excess neurotransmitter level. Figure 2A shows the times to steady state and steady state excess glutamate levels for all glutamate synapses. We divided the synapses into four groups based on the median time to steady state and median steady-state excess glutamate level. This resulted in synapse groups that were (1) timely maintained with a strong control of excess glutamate (blue), (2) timely maintained but with a weak control of excess glutamate (yellow), (3) untimely maintained with a strong control of excess glutamate (orange), and (4) untimely maintained with a weak control of excess glutamate (purple). Here, “strong” and “weak” refer to the *relative* ability of eCB signaling to reduce excess neurotransmitter release on a continuous dimension. Quantitatively, the relative magnitude of the excess neurotransmitter at a steady state is therefore the distinction between strong and weak control.

Next, to determine what factors contributed to synapses ending up in each group, we projected the synapses with their group identifier into the space of driving excitatory firing rate and glutamate bound to AMPARs (a proxy for how efficacious a synapse is). We show that this projection of features of excess dynamics into the space of model parameters resulted in groups that were still largely maintained: Figure 2B. This suggests that the combination of a model synapse with many AMPARs, lots of glutamate to release, and with high driving excitatory input permits eCB signaling components to strongly control excess glutamate. On the other hand, a synapse with few AMPARs hinders this homeostatic control, regardless of the input.

Figures 2C,D shows the results of the same analysis performed on GABA synapses and reveals that, unlike glutamate, the efficacy of a GABA synapse and the driving firing rate do not correlate with time to or final steady state GABA excess.

**Figure 3:**
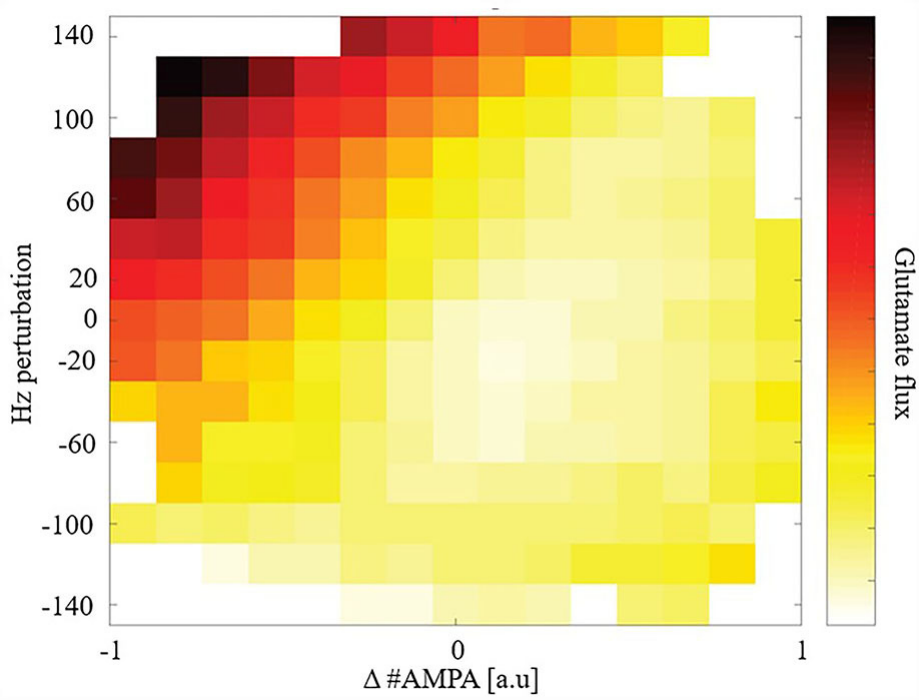
Glutamate flux given perturbations of synaptic weight and/or driving input.

Given the observed steady-state maintenance of glutamate release, next we conducted perturbation experiments. We focused our study on total glutamate cycled through release and uptake (glutamate flux) after a perturbation due to its high metabolic demand for removal and role in excitotoxicity. We perturbed the model to explore how synaptic plasticity or changes in driving excitatory input affects glutamate flux. We perturbed either, or both, the number of AMPARs and the driving excitatory firing rate for individual synapses to replicate changes due to synaptic plasticity or cortical activity changes and measured glutamate flux from the time of perturbation to the subsequent new steady state: Figure 3.

Synapses that underwent either LTD or received greater cortical input (higher firing rates) had a large glutamate flux. In comparison, synapses that either underwent long-term potentiation (LTP) or received reduced cortical input (lower firing rates) had a relatively smaller glutamate flux. Consequently, when LTD and an increase in cortical input were combined, we observed a very large glutamate flux among the model's synapses.

**Figure 4:**
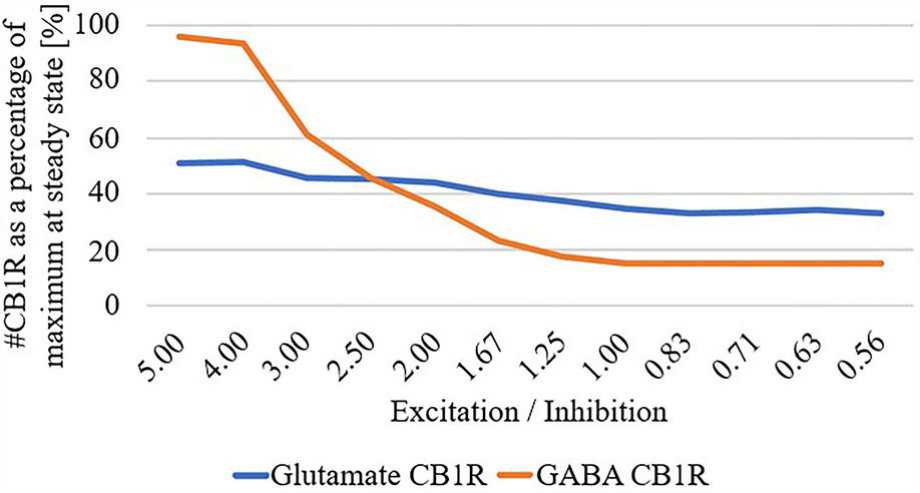
# Cannabinoid receptor (CB1R) as a percentage of maximum at steady state for glutamate and GABA as a function of excitation/inhibition ratio.

Finally, due to the observed disparate changes in eCB and widespread disconnect of excitatory input to the striatum in HD, we parameterized the total summation of all excitatory and inhibitory inputs at the network level, and systematically adjusted these inputs while quantifying the number of CB1R as a percentage of maximum in the unparameterized model: Figure 4. We found that as the excitatory/inhibitory ratio decreases, the numbers of both glutamatergic CB1Rs and GABAergic CB1Rs decrease, however, this reduction is markedly more prominent in GABAergic.

## Discussion

Our model predicts that eCB signaling can homeostatically maintain excess glutamate but not GABA. This is due to the different prerequisites for eCB production in the spine and dendrite in the model. In the spine, excitatory input drives all prerequisites for eCB production—mGluR5, NMDAR, and Ca^2+^ increases, while in the dendrite, GABA decreases the probability of its single prerequisite—Ca^2+^ increase. Furthermore, our model predicts that deficient/augmented eCB signaling might affect glutamatergic synapses and pathways more than GABAergic.

The excess amount of glutamate and its dynamics are potential risk factors for HD neurodegeneration. For example, if excess glutamate is too large, or the dynamics are too slow, the potential exists for glutamate to build up in the synaptic cleft. Consequently, due to the increased metabolic demand for uptake and metabolism, neurons and astrocytes may be placed under further stress, which if not met, may lead to excitotoxicity.

Results of our perturbation study predict that synapses that have a propensity to undergo LTD will transiently have large amounts of glutamate released. If the cortical input to these synapses fluctuates and transiently or tonically increases, this glutamate flux increases even more. Excess toxic glutamate has been suggested as a component of HD pathology (Liévens et al., 2001; Estrada-Sánchez and Rebec, 2012; Chiarlone et al., 2014) and our model suggests that the dynamics of eCB signaling may contribute. An interesting extension to this work can explore changes to the included effect of neurotransmitter transporters on the clearance of glutamate and GABA from the synaptic cleft.

Furthermore, as excitatory pathways degenerate and disconnect (Barry et al., 2021), the system may need to compensate for this loss of excitation. Our modeling results suggest that eCB signaling may asymmetrically decrease CB1R numbers on inhibitory terminals more than excitatory ones and therefore increase inhibition. While this is the opposite of a homeostatic response, if a disconnect of excitatory input is typically accompanied by dysfunction in glutamate uptake leading to excitotoxicity (Liévens et al., 2001; Estrada-Sánchez and Rebec, 2012), an increase in inhibitory tone is appropriate. This prediction of the model is based on (i) our assumption that eCB signaling is intact before the onset of HD symptomatic changes in eCB signaling and (ii) the inclusion of our proposed process *X*, which functions only within GABAergic boutons [the process mimics GABA insensitivity to eCB (Chiodi et al., 2011)]. The compensatory hypothesis for explaining the loss of CB1Rs in HD (Plotkin and Surmeier, 2015) was difficult to model in our hands, and so we view this extension of the model with a proposed process *X* as the most parsimonious solution, given our experiments with the model. For this reason, process *X* represents a prediction that will require additional focused experiments and investigation for testing. Future work could address the inverse cause and effect, i.e., changes to endocannabinoid signaling potentially changing glutamatergic or GABAergic signaling. Overall, substantial evidence supports dysregulated eCB signaling in HD mice mouse models. Therefore, eCB signaling presents several candidates for therapeutic targets. Chronic application of CB1R agonist in R6/1 mice prevented motor deficits and striatal MSN loss (Pietropaolo et al., 2014), genetic rescue of CB1R in R6/2 mice (with 150 CAG repeats) prevents the loss of excitatory input to the striatum (Naydenov et al., 2014), and environmental enrichment upregulates CB1R receptors and slows disease progression (Glass et al., 2004). GLT1 has been suggested as a pharmacological target (Soni et al., 2014) with its upregulation having been observed to attenuate HD phenotypes (Miller et al., 2008, 2012). Cannabidiol is also being explored as a clinical intervention for neurodegenerative disorders (Fernández-Ruiz et al., 2012). Due to the complex interaction between excitatory and inhibitory input and subsequent production and release of eCB, our modeling results suggest that therapeutics that target CB1R specifically on glutamatergic terminals may be needed, because given an overall decrease in the excitatory/inhibitory balance, increasing eCB signaling, in general, may counterproductively increase inhibitory tone.

## Materials and methods

### Network

**Figure 5:**
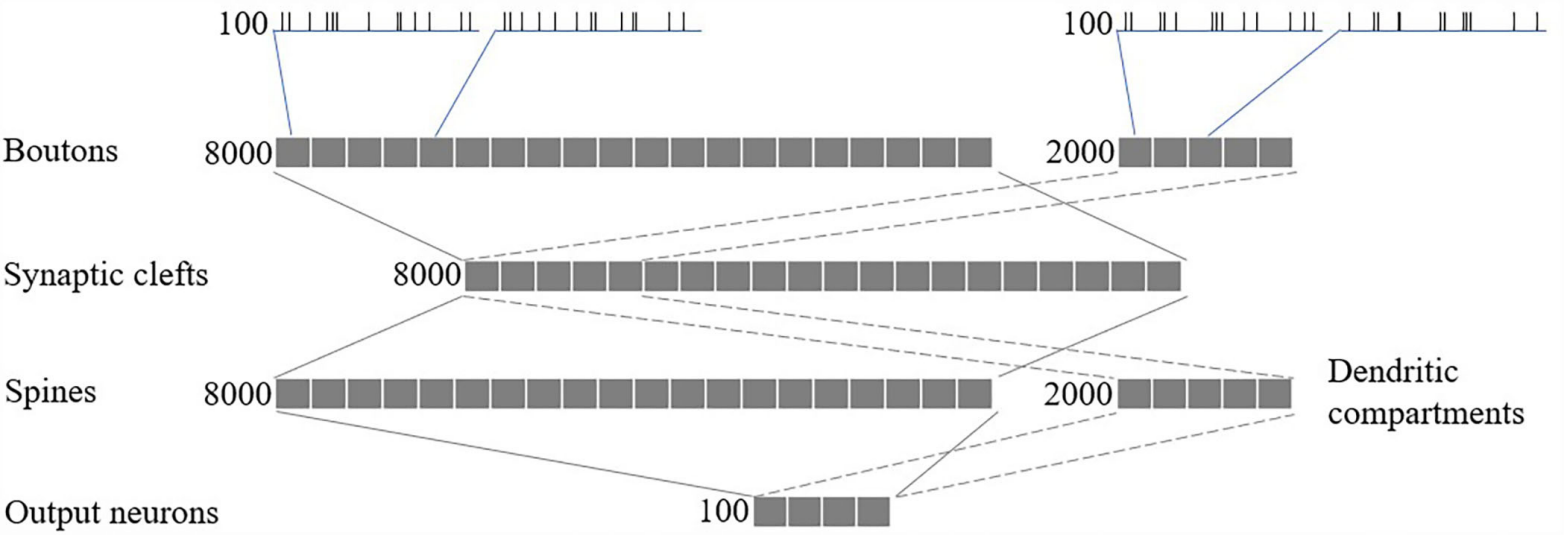
Network structure.

We modeled a network of 100 spiking neurons and their 100 excitatory and 100 inhibitory inputs (as shown in Figure 5). Synapses, comprising 8,000 glutamatergic boutons, 8,000 synaptic clefts, and 8,000 spines, and 2,000 GABAergic boutons and 2,000 dendritic compartments, were randomly connected between the inputs and neurons, such that each glutamatergic/GABAergic pathway received spikes from one random excitatory/inhibitory input. Finally, each output neuron has 80 randomly selected excitatory spines and 20 randomly selected inhibitory dendritic compartments.

### Synaptic space

**Figure 6:**
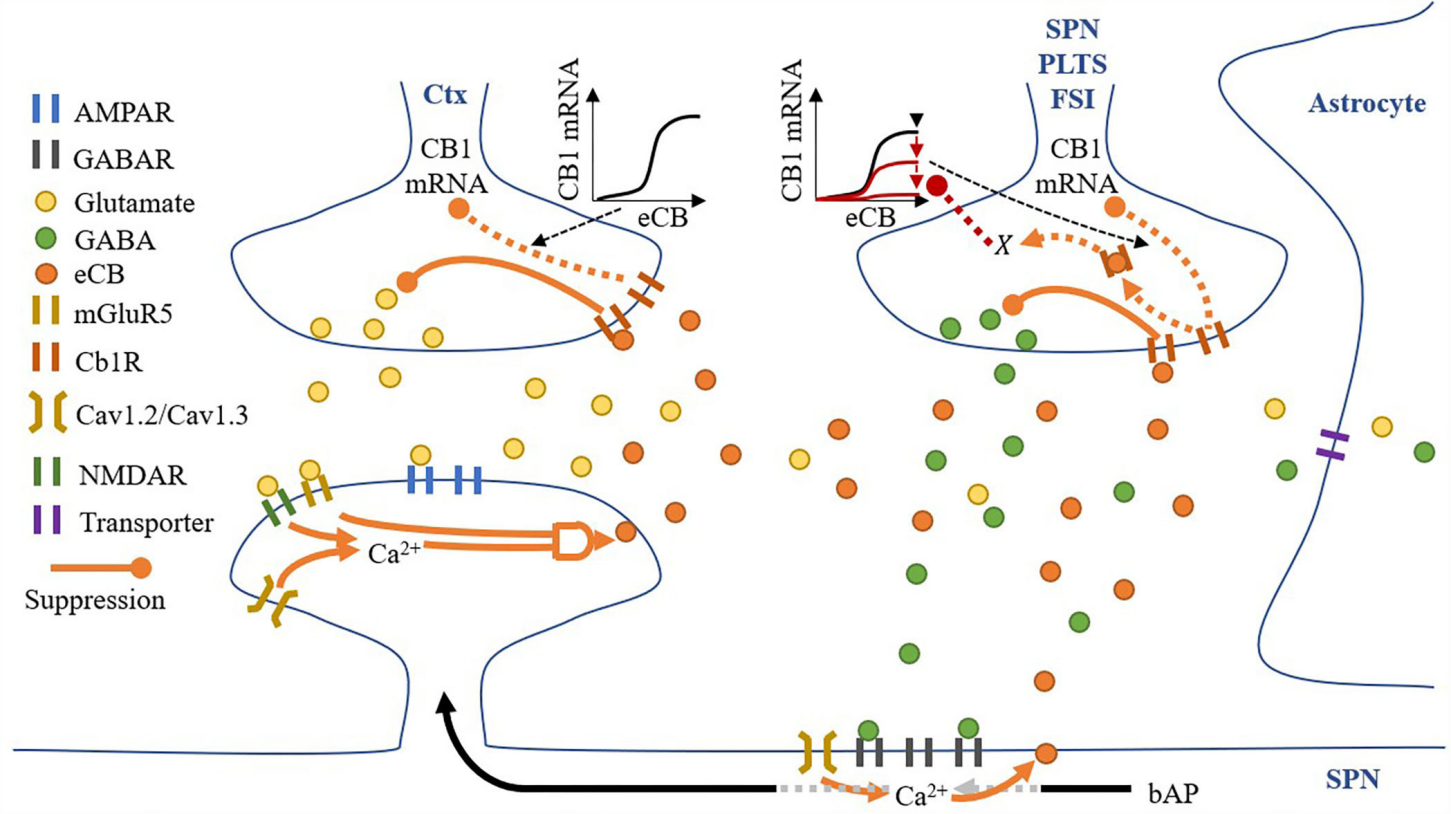
A synaptic space model. Glutamatergic and GABAergic terminals include neurotransmitter and eCB pathways.

All synaptic spaces in the network have neurotransmitter and eCB signaling pathways (as shown in Figure 6). Some synaptic spaces have only glutamatergic boutons and spines (6,000). Others have both glutamatergic boutons and spines and GABAergic boutons and dendritic shaft compartments (2,000).

Pre-synaptic neurons are not explicitly modeled—instead, all excitatory and inhibitory boutons receive spikes from independent homogenous Poisson processes.

Given a pre-synaptic spike, boutons release the available amount of their neurotransmitter, glutamate, or GABA, into the synaptic cleft. Cannabinoid receptors (*CB*1*R*) suppress the release of their neurotransmitter given activation by eCB in the synaptic cleft (either from the spine or dendritic compartment with different proportions), and in the absence of eCB, the number of neurotransmitters recovers to a set maximum amount. Each bouton also uses a modified Goodwin model (Gonze and Abou-Jaoudé, 2013) to include an observed relationship between eCB and CB1R mRNA (Laprairie et al., 2013). Specifically, unbound CB1R (*CB*1*R*_*unbound*_**) **reduces CB1R mRNA *via* a transcriptional inhibitor to limit the overall CB1R number (*#CB*1*R*). The motivational assumption is as follows: it is energetically inefficient to maintain many unused CB1Rs, so their number can signal the reduction in available CB1R mRNA and therefore limit CB1Rs. In addition, GABA boutons include our proposed process, *X*, corresponding to CB1R activation, with suppression of the maximum level of CB1R mRNA *via* the Goodwin model's *k*_1_. The motivational assumptions are as follows: (1) if the post-synaptic neuron is too active, high amounts of eCB will be present in the synaptic cleft (as shown in the dendritic compartment description below), and a desirable homeostatic response may release more GABA; and (2) *X* is an indirect measure of (1). Thus, if *X* suppresses the maximum level of CB1R mRNA, it will reduce CB1R suppression of GABA (Chiodi et al., 2011) and achieve the desired response. This causal relationship may play out in reality through any one or more of many biological pathways, so in the model, we simply assume that the relationship exists and model it directly.

Each synaptic cleft accumulates released glutamate and GABA, which is then reduced by neurotransmitter transporters. While the dynamics of these transporters are not directly modeled, their effective clearance of glutamate and GABA from the synaptic cleft is captured by the model. The model also contains eCBs from both the spine and dendritic compartment (if present) that does not accumulate. The eCBs that are released from the spines and dendritic compartments diffuse instantaneously to the glutamatergic and GABAergic boutons, respectively. However, there is some cross talk: some eCBs released from the spines activate CB1Rs on the GABAergic bouton and vice versa. Therefore, we include two local domains of eCBs, one for the spine (*eCB*_spine_) and one for the dendritic compartment (*eCB*_dendritic compartment_). Then, if a synapse has both glutamatergic and GABAergic boutons, crosstalk can be considered, but if there is only a glutamatergic bouton, just the spine's local domain is considered.

Spines process the release of glutamate by boutons and the subsequent presence of glutamate in the synaptic cleft in several ways: (1) instantaneous release of glutamate by the bouton activates AMPARs; (2) any excess glutamate which does not activate AMPARs activates mGluR5s; (3) NMDARs activation in response to a back-propagating spike, and proportionally to their open factor, which in turn is determined by the difference between the released glutamate and the amount of glutamate in the synaptic cleft. Following the activation of these receptors, spine *Ca*^2+^ concentrations increase *via* voltage-sensitive calcium channels (VSCCs) due to the depolarization of the spine by both AMPARs and NMDARs (each contributing 50% to the depolarization amount) and the size of the spine (proportional to the number of AMPARs). *Ca*^2+^ influx is dependent on the number of VSCCs and therefore surface area (assuming a linear relationship). Any change in *Ca*^2+^ concentration is then dependent on spine volume. The relationship between *Ca*^2+^ influx and spine volume is non-linear, and for simplicity we modeled this as *A*′. Finally, when *Ca*^2+^ levels are sufficient, *and* mGluR5s are active, eCB is produced on-demand in an amount proportional to both pre-requisites and released into the synaptic cleft.

A dendritic compartment is modeled only when a synapse has a GABA bouton. GABARs are activated by the instantaneous release of GABA by boutons, and local dendritic *Ca*^2+^ concentrations increase *via* VSCC due to the depolarization of the compartment by back-propagating spikes. In contrast to spines, in the dendritic compartments, the concentration of *Ca*^2+^ alone permits the on-demand production and release of eCB into the synaptic cleft.

Finally, each post-synaptic neuron integrates all its synapses' AMPARs, NMDARs, and GABARs, and if the membrane potential reaches threshold, fires, thereby producing a back-propagating action potential.

### Components

**Figure 7:**
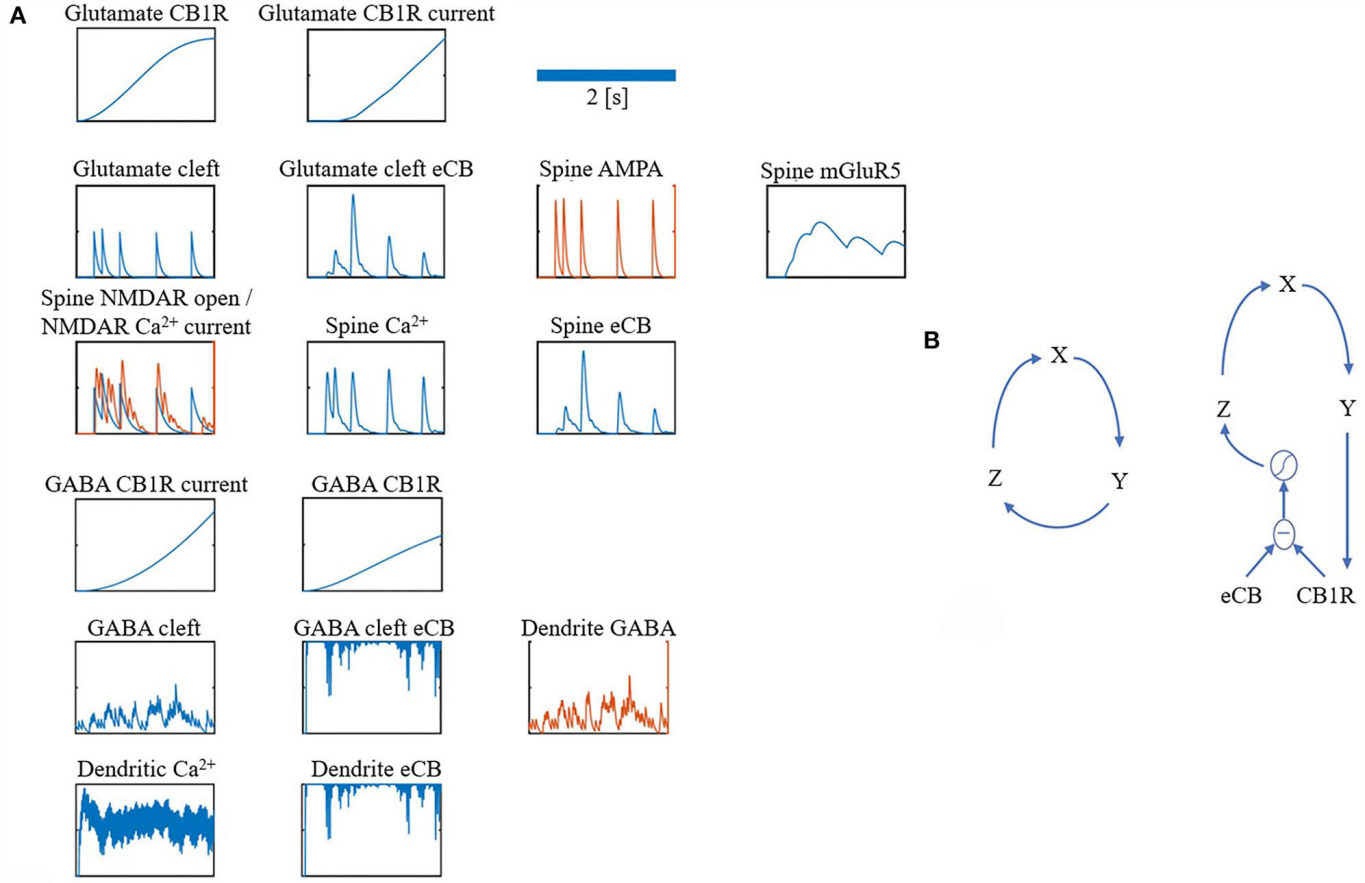
**(A,B)** Overview of model components traces.

For clarity, the notation ・ is used to represent a multiplication. Many of the components in the model share the same equations, however, they differ based on parameters, such as time constants or input. Therefore, all components are grouped into just four different forms: alpha function form (α), recovery form (β), leaky increase/accumulation form (γ), and adaptation form (φ) (as shown in Table 1 for a summary). In addition, some components have unique equations, which are listed under Section Other and some components combine more than one form. Figure 7A depicts traces for the main components.

**Table 1:** Summary of model component form.

**Component**	**α form**	**β form**	**γ form**	**φ form**	**Other**	**Bounded by ∈**
Glutamate/GABAs bouton's available glutamate/GABA		√		√		[0, 2]
Glutamate/GABA bouton's CB1R	√					[0, ∞)
Glutamate/GABA Goodwin model's *G*_*X*_, *G*_*Y*_, and *G*_*Z*_					√	[0, ∞)
Glutamate/GABA bouton's *CB*1*R*_*unbound*_					√	[0, 1]
Glutamate/GABA bouton's *#CB*1*R*					√	[0, 1]
GABA bouton's *X*	√					[0, ∞)
GABAs Goodwin model's *k*_1_		√		√		[0, 10]
Synaptic cleft's glutamate			√			[0, ∞)
Synaptic cleft's GABA			√			[0, ∞)
Synaptic cleft's spine's local domain's *eCB*					√	[0, ∞)
Synaptic cleft's dendritic compartment's local domain's eCB					√	[0, ∞)
Spine's AMPAR	√					[0, ∞)
Spine's mGluR5	√					[0, ∞)
Spine's NMDAR	√					[0, ∞)
Spine's *P*(*NMDAR*_*open*_)			√			[0, 1]
Spine's *Ca*^**2+**^	√					[0, ∞)
Spine's *eCB* instantaneous production					√	[0, 1]
Dendritic compartment's GABAR	√					[0, ∞)
Dendritic compartment's *Ca*^2+^	√					[0, ∞)
Dendritic compartment's *eCB* instantaneous production					√	[0, 1]

#### **α** form

General alpha function form for component α, where α′ is the rise current, α is the decay current, $\tau_{\alpha^{\prime }}$ and τ_α_ are the time constants for the rise and decay currents, respectively, and *I* is the input. Table 2 lists all parameters and *I*, *A*, and *A*′.


$$\begin{array}{lll} \tau_{\alpha^{\prime }}\frac{d\alpha^{\prime }}{dt} & = & I\\ \tau_{\alpha }\frac{d\alpha }{dt} & = & \alpha^{\prime } \end{array}$$

*A*: *if* [*Excitatory pre spike*·*Bouton*′*s available glutamate*]
>*#AMPAR*
then ([*Excitatory pre spike*·*Bouton*′*s available*
*glutamate*]−*#AMPAR*)·Ω_*mGluR*5_
*else* 0
*A*′: $\left(AMPAR\cdot \left[\Omega_{Spine}^{VSCC}\cdot \#AMPAR^{-0.5}\right]\right)+$

$\left(NMDAR\cdot \Omega_{NMDAR}^{Ca^{2+}}\right)$

**Table 2:** Table of α form parameters.

	**Component α**	**τ_α_^′^(s)**	**τ_α_ (s)**	**Input I (a.u.)**
Glutamate bouton	Glutamate's CB1R	4	22.5	arg min(*eCB*_*glutamate*_ , *#CB*1*R*) see Section α form.
GABA bouton	GABA's CB1R	4	22.5	arg min(*eCB*_*GABA*_ , *#CB*1*R*) see Section α form.
	*X*	4	22.5	GABA's CB1R ·ω_*CB*1*R*→*X*_
Spine	AMPAR	0.004	0.030	arg min$\left(\left[Excitatory\;pre\;spike\cdot Bouton^{\prime }s\;available\;glutamate\right]\;,\;\#AMPAR\right)\cdot \Omega_{AMPAR}$
	mGluR5	0.25	0.25	*A*
	NMDAR	0.02	0.10	Post spike ·*P*(*NMDAR*_*open*_)·Ω_*NMDAR*_
	*Ca* ^2+^	0.010	0.008	*A*′
Dendritic compartment	GABAR	0.0008	0.0130	arg min$\left(\left[Inhibitory\;pre\;spike\cdot Bouton^{\prime }s\;available\;GABA\right]\;,\;\#GABAR\right)\cdot \Omega_{GABAR}$
	*Ca* ^2+^	0.010	0.008	$Post\;spike\cdot \Omega_{Dendritic\;compartment}^{VSCC}$

### **β** form

General form for the recovery of component β to some maximum value. Parameters are shown in Table 3.


$$\begin{array}{l} \tau_{\beta }\frac{d\beta }{dt}=\beta_{\max}-\beta \end{array}$$

**Table 3:** Table of β form parameters.

**Component β**	**τ_β_ (s)**	**β_max_** (*a*.*u*.)****
Available glutamate/GABA	4.28	2
GABA's Goodwin model's *k*_1_	4.28	10

### **γ** form

General form for the leaky increase/accumulation of component γ with time constant τ_γ_ and input *I*. Table 4 lists parameters and *I*.


$$\begin{array}{l} \tau_{\gamma }\frac{d\gamma }{dt}=-\gamma +I \end{array}$$

**Table 4:** Table of γ form parameters.

**Component γ**	**τ_γ_ (s)**	**Input I (a.u.)**
Synaptic cleft's glutamate	0.045 [fit to (Diamond, 2005)]	Excitatory pre spike · Bouton's available glutamate
Synaptic cleft's GABA	0.005	Inhibitory pre spike · Bouton's available GABA
Spine's **P**(*NMDAR*_*open*_)	0.100	*ifSynaptic cleft*′*s glutamate* −(*Excitatory pre spike* ·*Bouto*′*s available glutamate*)>0 *thenSynaptic cleft*′*s glutamate* −(*Excitatory pre spike* ·*Bouton*′*s available glutamate*) *else*0

### **φ** form

General form for adaptation of component ε. Table 5 lists parameters.


$$\begin{array}{l} \frac{d\varepsilon }{dt}=Z\cdot \varphi \end{array}$$

**Table 5:** Table of adaptation of component ε parameters.

**Rate**	**Z (a.u.)**	**φ (a.u.)**
Available glutamate / GABA	arg min[*Synaptic cleft*′*s* (*glutamate*′*s*/*GABA*′*s*) *eCB* , *#CB*1*R*]	−0.00005 (a.u)
**k**_**1**_	*X*	−0.0001 (a.u.)

### **ω** / **Ω**

Weight ω_α → β_ for connection between component α and β, and component weight Ω. Table 6 lists parameters.

**Table 6:** Table of ω weights.

**α**	**β**	**ω (a.u.)**
**G**_**Y**_	#CB1R	0.0067
CB1R	*X*	5
CB1R_unbound_	*G*_*Z*_	400
**Description**	**Ω**	**Value**
AMPAR weight	Ω_*AMPAR*_	1.28 (a.u.)
mGLuR5 weight	Ω_*mGluR*5_	1600 (a.u.)
NMDAR weight	Ω_*NMDAR*_	0.32 (a.u.)
Spine's VSCC weight	$\Omega_{Spine}^{VSCC}$	125 (a.u.)
NMDAR's *Ca*^2+^ weight	$\Omega_{NMDAR}^{Ca^{2+}}$	800 (a.u.)
Dendritic compartment's VSCC weight	$\Omega_{Dendritic\;compartment}^{VSCC}$	0.06 (a.u.)
GABAR weight	Ω_*GABAR*_	−1.6 (a.u.)

### Other


$$\begin{array}{l} \;\#CB1R=G_{Y}\cdot \omega_{G_{Y}\to \#CB1R}\\ \;CB1R_{unbound}=\#CB1R-Synaptic\;cleft^{\prime }s\\ \;\left(glutamate^{\prime }s/GABA^{\prime }s\right)\;eCB\\ \;S\left(x,C,D\right)=\frac{1}{1+e^{-C\cdot \left(x-D\right)}}\\ S^{\prime }\left(x^{\prime },C^{\prime },D^{\prime }\right)=\left(S\left[x^{\prime },C^{\prime },D^{\prime }\right]-S\left[0,C^{\prime },D^{\prime }\right]\right)\\ \;.\left(\frac{1}{S\left[1,C^{\prime },D^{\prime }\right]-S\left[0,C^{\prime },D^{\prime }\right]}\right) \end{array}$$

Instantaneous spine and dendritic compartment eCB production:


$$\begin{array}{l} eCB_{\text{spine}}=S^{\prime }(Spine^{\prime }s\;Ca^{2+}\\ \;.S^{\prime }\left[mGluR5\;,\;eCB_{C}\;,\;eCB_{D}\right]\;,\\ \;eCB_{C}\;,\;eCB_{D})\\ eCB_{\text{dendritic compartment}}=S^{\prime }(Dendritic\;compartment^{\prime }s\;Ca^{2+},\\ \;\;eCB_{C}\;,\;eCB_{D}) \end{array}$$

If a synapse has a glutamatergic and GABAergic bouton:


$$\begin{array}{lll} eCB_{\text{glutamate}} & = & eCB_{\text{spine}}\cdot \left(1-cross\right)\\ +eCB_{\text{dendritic compartment}}\cdot cross\\ eCB_{GABA} & = & eCB_{\text{dendritic compartment}}\cdot \\ \left(1-cross\right)+eCB_{\text{spine}}\cdot cross \end{array}$$

If a synapse only has a glutamatergic bouton:


$$\begin{array}{lll} eCB_{\text{glutamate}} & = & eCB_{\text{spine}}\\ eCB_{GABA} & = & 0 \end{array}$$

### Additional parameters

Table 7 lists additional parameters.

**Table 7:** Table of additional parameters.

**Gain description**	**Parameter**	**Value**
Amount of eCB crosstalk in the synaptic cleft	*cross*	0.25
Spine's/Dendritic compartment's eCB **S**() parameters	*eCB*_*C*_ and *eCB*_*D*_	10 (a.u.) and 0.5 (a.u.)

### Goodwin model

For both the glutamate and GABA terminals' Goodwin model (Gonze and Abou-Jaoudé, 2013), a protein *G*_*Y*_, where *G*_*X*_ is its mRNA, and *G*_*Z*_ is its transcriptional inhibitor, where *S* is the sigmoid function. This closed loop form is a parsimonious description of the interaction between a protein and its mRNA and transcriptional inhibitor (as shown in Figure 7B left).

Instead of having a complete interaction between CB1Rs (protein) and their transcriptional inhibitor as in the classical model, we restricted the interaction to only unbound CB1Rs:


$$\begin{array}{lll} \tau_{G}\frac{dG_{X}}{dt} & = & \frac{k_{1}}{K_{1}+{G_{Z}}^{n}}-k_{2}\cdot G_{X}\\ \tau_{G}\frac{dG_{Y}}{dt} & = & k_{3}\cdot G_{X}-k_{4}\cdot G_{Y}\\ \tau_{G}\frac{dG_{Z}}{dt} & = & k_{5}\cdot S\left({CB1R}_{unbound}\cdot \omega_{{CB1R}_{unbound}\to G_{Z}}\;,\;G_{C}\;,\;G_{D}\right)\\ -k_{6}\cdot G_{Z} \end{array}$$

Figure 7B (right) shows a depiction of this restricted interaction between unbound CB1Rs (the difference between eCB and CB1Rs) and their transcriptional inhibitor (*Z*). For parameters refer to Table 8.

**Table 8:** Table of Goodwin model parameters.

τ_G_	20 (s)
*K*_1_	1 (a.u.)
Glutamate's *k*_1_	10
GABA's *k*_1_	See β and φ forms
*k*_2_	1 (a.u.)
*k*_3_	15 (a.u.)
*k*_4_	1 (a.u.)
*k*_5_	15 (a.u.)
*k*_6_	0.001 (a.u.)
*G*_*C*_	0.3 (a.u.)
*G*_*D*_	50 (a.u.)

### Change to global excitatory and inhibitory weights

To change the excitatory/inhibitory ratio, global weights were included for the excitatory and inhibitory inputs. These were systematically adjusted, and the number of CB1R as a percentage of maximum in the unweighted model quantified.

### Initial conditions

At the beginning of a simulation α and γ form, components are set to 0, and β and φ forms and *#CB*1*R* are set to a random value Uniformly chosen within their bounds.

## Data availability statement

The original contributions presented in the study are publicly available. This data can be found here: https://github.com/JamesHumble/Humble-Kozloski-Cannabinoid-Signaling. The network model was implemented in IBM Model Graph Simulator, which is the core parallel processing architecture for model description (using the Model Description Language, MDL) and resource allocation (using the Graph Specification Language, GSL) of the Neural Tissue Simulator (Kozloski and Wagner, 2011). The Model Graph Simulator software is experimental. Readers are therefore encouraged to contact the authors if interested in using the tool.

## Author contributions

JH and JK designed and performed research and wrote the paper. Both authors contributed to the article and approved the submitted version.

## Funding

This work was supported partly by the CHDI Foundation.

## Conflict of interest

The authors declare that the research was conducted in the absence of any commercial or financial relationships that could be construed as a potential conflict of interest.

## Publisher's note

All claims expressed in this article are solely those of the authors and do not necessarily represent those of their affiliated organizations, or those of the publisher, the editors and the reviewers. Any product that may be evaluated in this article, or claim that may be made by its manufacturer, is not guaranteed or endorsed by the publisher.

## Footnotes

^1^Activation of mGluR5s is a common prerequisite for eCB signaling.
